# Effect of pre-fabricated strip, zirconia and stainless steel crowns in primary molars

**DOI:** 10.6026/973206300191388

**Published:** 2023-12-31

**Authors:** Shalini Singh, Arpanna Singh, Subhranshu Sekhar Sahoo, Chandan Singh, Indranil Mukhopadhyaya, Aum Joshi

**Affiliations:** 1 Department of Dentistry, School of Dental Sciences, Sharda University, Greater Noida, Uttar Pradesh, India; 2 Department of Pedodontics and Preventive Dentistry, Community Health Center, Health and Family Welfare, Ghazipur, Uttar Pradesh, India; 3 Department of Pedodontics and Preventive Dentistry, SCB Dental College and Hospital, Cuttack, Odisha, India; 4 Pedodontics and Preventive Dentistry, Private Practitioner, Cap and Crown Family Dental Clinic, Patna, Bihar, India; 5 Oral-Dental Surgeon and Oral Medicine Specialist, Laser Surgeon, Apollo Clinic Thakurpukur, Kolkata, West Bengal, India; 6 Department of Pedodontics and Preventive Dentistry, College of Dental Sciences, Amargadh, Gujarat, India

**Keywords:** Zirconia crowns, resin strip crowns, stainless steel crowns

## Abstract

The effect of gingival, clinical and radiographic outcomes while using prefabricated stainless steel crowns (SSC), resin strip crowns and zirconia crowns in primary molars is of interest to dentists. Clinical periodontal and radiographic evaluation was conducted for three groups at baseline 3, 6, 9 and 12 months intervals utilizing scoring system. According to the current study's findings, gingival health was better in the zirconia crown group compared to the resin strip crown and SSC groups. In addition, zirconia group demonstrated respectable clinical and radiographic outcomes when compared to resin strip crowns and stainless steel crowns, with the added benefit of better aesthetics.

## Background:

Extensive carious lesions involving more than one tooth surface in the deciduous dentition suggest a complete tooth covering restoration [1,2,3, 4]. Additionally, it should be used in cases of circumferential tooth decay, profound tooth decay either bilaterally or unilaterally and tooth with history of endodontic therapy [5, 6,7]. In 1950, Dr. William Humphrey introduced the concept of stainless steel crowns (SSC). When it came to complete coverage, these seemed to the most dependable restoration [8, 9,10]. SSC was the preferred course of action following pulpectomy or pulpotomy since it had less microleakage than amalgam restorations. Unlike composite resin crowns, SSCs don't need to be completely isolated in order to bond, and unlike amalgam restorations, they don't need to be prepared by adding mechanical retention modifications into the design [11-12]. The overall incidence of SSC perforations after a two-year period of clinical use was a mere 12%. Nonetheless, the emergence of metal-free covering is a result of the parents' desire for realistic restorations that resemble natural teeth [13, 14,15]. The application of zirconia crowns, which are regarded as a cosmetic procedure in comparison to other crown alternatives, serves as an example [4- 7]. Through the use of computer assisted design (CAD) and computer assisted machining (CAM), zirconia ceramics that were created from a single material were able to increase their translucency, resulting in outstanding mechanical characteristics and flawless aesthetic crowns [8-10].

In primary molars, orthodontic treatment and early childhood caries frequently cause the dental crown framework to be lost. Early tooth loss has negative consequences, including speech difficulties, space closure, and the practice of pushing the tongue forward and psychological repercussions [12-14]. The dentist's job is never easy when it comes to restoring teeth that are so badly damaged and involve the pulp. Numerous aesthetic methods of treatment have been proposed for the treatment of tooth decay and injury in the deciduous dentition [15, 16,17]. Full anatomic aesthetic restorations-resin composite strip crowns, prefabricated crowns such as pre-veneered SSCs, recently developed a ready-made primary zirconia crowns-are recommended for the rehabilitation of deciduous teeth [4-8]. Crowns made of resin strips are frequently used on primary anterior teeth. The following are some of the benefits of strip crowns: excellent aesthetics, ease of application, the ability to be repaired, and high patient as well as parent approval [19]. Technique sensibility, the necessity for sufficient dental structure for mechanical retention, cooperation from patients, and the increased likelihood of breakage under trauma are some of its drawbacks [20,21, 22,23,24, 25].

Many research works have examined the gingival condition of primary molars that have been restored using SSC. Healthy gingiva and reduced plaque accumulation were associated with well-to-moderately integrating crowns as well as well-contoured edges [13,17]. However, some researchers have linked variations in the sub-gingival margins of the SSC to inflammation in the gingival region following SSC restoration of a primary molar [5-9]. The examination of their clinical, periodontal and radiographic success as the final restoration of pulp optimized deciduous molars was made necessary by the debates surrounding SSCs, resin strip crowns, and zirconia crowns. Therefore, it is of interest to evaluate and compare gingival, clinical and radiographic outcomes while using prefabricated SSC, resin strip crowns and zirconia crowns in primary molars.

## Methods and Materials:

## Research subjects:

360 medically uninjured kids (188 boys and 172 girls) with 720 vital mandibular primary molars having age ranging from 4 year to 6 years old who had reported to the Department of Paediatric Dentistry were included in this research.

## Inclusion criteria:

[1] Deep carious lesions involving the first along with second deciduous molars on both sides were present in the patients.

[2] Lack of any indication of a clinical pathology

[3] Was immobile and showed no sensitivity to percussion

[4] A standard or no resorbed level of interproximal bone, where the radiographic assessment showed that the measurement between CEJ and the crest of interdental bone was not larger than 2 mm.

[5] Root resorption was found to be no greater than one third.

## Exclusion criteria:

[1] History of systemic disorders

[2] An intolerance to any medication, including those used as local anesthetics

[3] Exceptionally bad dental hygiene

[4] Any Dental disease

[5] Findings of malocclusion.

Patients received treatment while sedated. Following the administration of local anesthesia, all caries was taken out and the pulp chamber was exposed by coronal access obtained with a clean rapidly rotating bur number 330 that sprayed water. Coronal pulp removal was performed using a disinfected spoon excavator. Hemostasis was achieved after lightly pressing sterile cotton pellets dampened with distilled water for a period of five minutes that were laid over the pulp. The molar was removed from the study after five minutes, if the bleeding continued. By applying an uncontaminated cotton pellet, formo cresol (Dentsply, Surrey, UK) was placed for three to five minutes. The pulp stumps were covered with a base of reinforced zinc oxide eugenol following the removal of the cotton pellet. Following pulp therapy, molars underwent restoration and were split into three equal groups (Table 1).

## Group 1: Crowns made of stainless steel (control):

Uniform occlusal reduction by employing a flame-shaped diamond bur to reduce the occlusal surface by roughly 1.5 mm is completed. By applying a long, tapering diamond bur, interproximal slices were cut mesially as well as distally by adhering diamond bur marginally convergent. The probe should be able to move through the region of contact owing to the reduction. The prepared tooth's mesiodistal width was taken into consideration when selecting the proper size, and a trail fit was performed prior to cementation. The crown shouldn't stay subgingivally more than 1 mm. SSCs that were embellished and moulded were cemented.

## Group 2: Zirconia crowns:

NuSmile Try-In Crowns can be used to determine the appropriate crown size, which should always be chosen before beginning molar reduction. A 1-1.5 mm reduction in the occlusal surface was carried out adjacent to the typical occlusal profile. Opening of interproximal contacts took place. The selected crown should be able to fit passively in the proximal space. Using tapered diamond burs, the molar tooth should be embellished down circumferentially by 0.5-1.25 mm as needed. Coarse diamond burs in the shape of a football could be used to reduce the occlusal area.

## Sub gingival reduction:

In order to ensure that there will be no undercuts and subgingival ridges, the planned margin should be honed to a feather-edge, staying about 1-2 mm beneath the gums on every area. It is best to use a thin, tapered diamond bur to avoid shattering tissue when making subgingival tooth modifications. In order to enable a slight rounding of all preparation areas, line angle as well as point angles was finally eliminated.

## Group 3: Strip crown:

Strip crown was the preferred material for this category's coronal build-up. To create the crown, a suitable-sized strip crown was chosen. The tooth was reduced interproximally for 0.5-1.0 mm during tooth preparation for crown. With the aid of a finely tapered bur, the occlusal surface of tooth was decreased by 1.5 mm. One and five milli-metres were taken off the labial surfaces along with lingual surfaces, respectively. At the border of the gingival tissue, a feather edge was formed. The gingival margin was cut with scissors to conform the tooth with strip crown. After using a 37 percent phosphoric acid liquid to etch the surfaces of tooth for 15 seconds, they were carefully rinsed with water. Following air pressure drying of the surfaces of the teeth, a bonding agent layer was added. It underwent polymerization for a further twenty seconds. After inserting the celluloid crown filled with composite, extra resin was scraped off. The material underwent light curing, and the strip crown was removed. Occlusion was examined and modified as necessary. Finishing was carried out using burs and shofu discs. Clinical periodontal and radiographic evaluation were conducted for three groups at baseline, 3,6,9 and 12 months intervals utilizing Scoring system.

## Clinical success standards:

The following standards were used for the clinical evaluation of the crowns:

[1] Length: the margin extends to CEJ or stops at the gingival crest.

[2] Position: the crown is not turned.

[3] Smoothness: free of blemishes and rough edges.

[4] Cement: the sulcus contains no extra cement.

Clinically, a crown is deemed acceptable if all requirements are met; otherwise, it is deemed unacceptable.

## Radiographic evaluation:

Molars with crown were radio-graphically assessed at baseline, 3 months, 6 months, 9 months, and 12 months intervals using the periapical parallel approach. Kodak pediatric film size 0 and the RinnXCP film holder helped to achieve a standardized technique.

## Requisites of radiographic success:

The crown's quality is deemed adequate when all of its margins are seen flawless and well-adapted, encompassing all of the dentin. This is the first criterion for radiographic success. When defects are found in the crown, or when the margins of crown appear excessively short or went below CEJ or displaced from the surface of the tooth by a measurement greater than 1mm, the crown is deemed inadequate. 2. When the separation between CEJ and the highest point of the interdental bone is 2 mm or lower, the interproximal bone threshold is deemed normal with no resorbed. When the separation exceeds 2 mm, the bone is deemed resorbed.

## Periodontal Outcomes:

Gingival index (GI) [8-10] and Oral hygiene index [11-14] was used for assessment of gingival health and periodontal health.

## Statistical analysis:

By examining the distribution of the data and applying normalcy tests (Kolmogorov-Smirnov and Shapiro-Wilk tests), numerical data were examined for normality. While GI along with OHI scores was considered nonparametric data, age data displayed a parametric distribution. The information was displayed using the following metrics: mean median, standard deviation (SD), minimum, maximum, and 95% Confidence Interval (95% CI). The Student's t-test was employed to compare the two groups when dealing with parametric data. The Mann-Whitney U test was utilised to compare two groups' when dealing with non-parametric data. Friedman's test was employed to examine the temporal changes within each group. Pairwise comparisons between the intervals where Friedman's test is significant were performed using the Wilcoxon signed-rank test with Bonferroni's adjustment. Frequencies (n) and percentages (%) were used to present the qualitative data. The two groups were compared using the chi-square test. A significance threshold of P ≤ 0.05 was established. For statistical analysis, IBM® SPSS® Statistics Version 20 for Windows was used.

## Results:

On analyzing GI scores at different time durations it was observed that GI scores increased in all three types of crowns when there were evaluations within the group (p<0.001). The difference in GI scores among the three categories was statistically significant at 9 months and 12 months follow up. The GI score at baseline was comparable among three categories. The GI score at 12 month follow up in Zirconia group (0.38± 0.023) was low while GI score in SSC category (0.63±0.081) was high. The values of GI score in Strip crown (0.46 ± 0.012) were greater than zirconia group, but lesser than SSC. These values demonstrated that gingival health was better in Zirconia crowns (Table 2).

On analyzing OHI scores at different time durations it was observed that OHI scores increased in all three types of crowns when there was evaluations within the group (p<0.001). The difference in OHI scores among the three categories (intergroup comparisons) was statistically significant at 9 months and 12 months follow up. The OHI score at baseline was comparable among three categories. The OHI score at 12 month follow up in Zirconia group (0.39± 0.024) was low while GI score in SSC category (0.64±0.092) was high. The values of OHI score in Strip crown (0.47 ± 0.025) were greater than zirconia group, but lesser than SSC. These values demonstrated that oral hygiene was better in Zirconia crowns (Table 3).

While evaluating clinical success, it was observed that 75.7% crowns in SSC category were accepted at 12 month follow up, while 7.7% were non accepted and 16.25% crowns were not evaluated because of patient drop out. Similarly in Zirconia crowns category, proportion of acceptance was 80.9% while proportion of non acceptance was 5.9% and 12.5% were drop out. In case of Strip crown the acceptance percentage was 81.67, non acceptance percentage was 7.09% and drop out percentage was 11.25%. It was inferred that Zirconia crowns had maximum clinical success followed by strip crown and SSCs. (Table 4)

While evaluating radiological success, it was observed that 75.8% crowns in SSC category were accepted at 12 month follow up, while 7.3% were non accepted and 16.22% crowns were not evaluated because of patient drop out. Similarly in Zirconia crowns category, proportion of acceptance was 80.9% while proportion of non acceptance was 5.9% and 12.5% were drop out. In case of Strip crown the acceptance percentage was 81.68, non acceptance percentage was 7.02% and drop out percentage was 11.25%. It was inferred that Zirconia crowns had maximum radiological success followed by strip crown and SSCs (Table 5).

While evaluating interproximal bone loss, it was observed that interproximal bone loss in 74.17% crowns in SSC category were accepted at 12 month follow up, while 5.5% were non accepted and 16.67% crowns were not evaluated because of patient drop out. Similarly in Zirconia crowns category, proportion of acceptance was 79.17% while proportion of non acceptance was 7.5% and 13.34% were drop out. In case of Strip crown the acceptance percentage was 76.25, non acceptance percentage was 8.34% and drop out percentage was 15.42%. It was inferred that Zirconia crowns had maximum accepted interproximal bone loss. This is followed by strip crown and SSCs at 12 month follow up (Table 6). It can be inferred that clinical success, radiological success and accepted interproximal bone loss was maximum in zirconia crowns followed by strip crowns and SSCs. However, the difference was not significant statistically.

## Discussion:

Speech problems, space closure, the habit of thrusting the tongue forward, and psychological effects are all associated with early tooth loss. When it comes to repairing teeth that are severely damaged and involve the pulp, the dentist's job is never simple [16]. The discussions around SSCs, resin strip crowns, and zirconia crowns necessitated an analysis of their clinical, periodontal, and radiographic results as the last restoration of pulp-otomized deciduous molars. Zirconia crowns' highly polished, smooth surfaces may be to blame for this, as they reduced plaque accumulation and the ensuing gingival irritation [16,17]. Positive gingival health was observed in primary anterior Zirconia crowns, according to a different study by other researchers [18- 20]. However, another study showed that incorrectly shaped metal borders and adhesive remnants in the gingival sulcus in a case of SSCs were major sources of gingival irritation, leading to additional plaque accumulations and ensuing gingival inflammation [19-23]. Numerous studies have looked at the gingival health of primary molars that have undergone SSC restorations. Well-to-moderately integrating crowns and well-contoured edges were linked to healthy gingiva and less plaque buildup [8- 14].Nonetheless, following SSC restoration of a primary molar, some researchers have connected variations in the sub-gingival margins of the SSC with gingival inflammation [12- 17]. SSCs don't require complete isolation to bond, unlike composite resin crowns, and they don't require mechanical retention adjustments to be made to the design, unlike amalgam restorations [14- 19]. Zirconia crowns in our study had a higher success rate, which may have been caused by their superior corrosion resistance, exceptional durability, outstanding mechanical properties, high flexure strength, smooth and glossy surface and biocompatibility. The current study's findings while not statistically significant, demonstrated that zirconia crowns outperformed strip crowns in terms of fracture toughness, lack of reliance on the remaining tooth structure for retention, and reduced polymerization shrinkage. The current study found that zirconia crowns outperformed the other two types of crowns, but because of their high cost, middle-class people still find it difficult to afford them. In these situations, resin strip crowns can be used as an alternative restoration method for primary molars. Conversely, previous studies carried out a two-year randomized control trial to examine primary tooth restoration. Ninety-five percent of restored teeth with metal crowns survived. The success of zirconia crowns on primary molar teeth is currently not well documented, with the exception of research conducted by the product's manufacturer, NuSmile ZR, located in Houston, Texas, USA [20-24]. After two years of clinical use, the overall incidence of SSC perforations was only 12% [4- 7]. However, the parents' desire for lifelike restorations that mimic natural teeth is what led to the development of metal-free covering. One example of this is the use of zirconia crowns, which are considered a cosmetic procedure when compared to other crown alternatives [5-8]. Zirconia ceramics, which were made from a single material, were able to increase their translucency through the use of computer assisted design (CAD) and computer assisted machining (CAM). This resulted in exceptional mechanical properties and faultless aesthetic crowns [16,17]. The results of this investigation supported previous study [26] findings, which established a direct link between interproximal bone resorption and stainless steel crowns. Specifically, the study indicated that crowns deemed non-satisfactory radiographically were linked to interproximal bone resorption. Furthermore, other studies reported that alveolar bone resorption and gingival inflammation have been reported significantly in SCCs in deciduous molars, particularly when insufficient crown reduction, position, contour, length and increased cement remnants were seen in the gingival sulcus [9,21].

## Conclusion:

Gingival health was better in the zirconia crown group compared to the resin strip crown and SSC groups. In addition, zirconia group demonstrated respectable clinical and radiographic outcomes when compared to resin strip crowns and stainless steel crowns, with the added benefit of better aesthetics.

## Figures and Tables

**Table 1:** Distribution of study participants

**Groups**	**Crowns**	**No of patients**	**No of primary molars**
Group 1	SSC	120	240
Group 2	Zirconia	120	240
Group 3	Resin strip	120	240

**Table 2:** Comparison of GI scores in three categories

	**Base line**	**3 months**	**6 months**	**9 months**	**12 months**	**P value (within group)**
SSC	0.01±0.00	0.05±0.002	0.10±0.004	0.35±0.02	0.63±0.081	<0.001
Zirconia	0.00±0.01	0.01±0.006	0.06±0.001	0.19±0.04	0.38± 0.023	<0.001
Strip Crown	0.00±0.02	0.03±0.002	0.08±0.006	0.24±0.03	0.46 ± 0.012	<0.001
P value	1	0.067	0.432	0.018*	0.012*	

**Table 3:** Comparison of OHI scores in three categories

	**Base line**	**3 months**	**6 months**	**9 months**	**12 months**	**P value (within group)**
SSC	0.01±0.01	0.06±0.003	0.11±0.05	0.36±0.03	0.64±0.092	<0.001
Zirconia	0.00±0.00	0.02±0.007	0.07±0.002	0.21±0.05	0.39 ±0.024	<0.001
Strip Crown	0.00±0.01	0.04±0.003	0.09±0.007	0.25±0.04	0.47 ± 0.025	<0.001
P value	1	0.067	0.432	0.018*	0.012*	

**Table 4:** Comparison between criteria of clinical success in the three categories

		**Base line**	**3 months**	**6 months**	**9 months**	**12 months**		
**Criteria**		**A**	**A**	**A**	**A**	**A**	**NA**	**D**
SSC	n	240	240	240	240	183	19	38
	%	100	100	100	100	75.7	7.7	16.25
Zirconia	n	240	240	240	240	195	15	30
	%	100	100	100	100	80.9	5.9	12.5
Strip Crown	n	240	240	240	240	196	17	27
	%	100	100	100	100	81.67	7.09	11.25
P value		NA	NA	NA	NA	0.658		
A= Acceptable; NA=Non acceptable; D= Dropout

**Table 5:** Comparison between criteria of radiological success in the three categories

		**Base line**	**3 months**	**6 months**	**9 months**	**12 months**		
**Criteria**		**A**	**A**	**A**	**A**	**A**	**NA**	**D**
SSC	n	240	240	240	240	184	18	38
	%	100	100	100	100	75.8	7.3	16.22
Zirconia	n	240	240	240	240	205	14	31
	%	100	100	100	100	80.9	5.9	12.5
Strip Crown	n	240	240	240	240	197	13	27
	%	100	100	100	100	81.68	7.02	11.25
P value		NC	NC	NC	NC	0.658		
A= Acceptable; NA=Non acceptable; D= Dropout

**Table 6:** Comparison between criteria of inter proximal bone loss in the three categories

		**Base line**	**3 months**	**6 months**	**9 months**	**12 months**		
**Criteria**		**A**	**A**	**A**	**A**	**A**	**NA**	**D**
SSC	n	240	240	240	240	178	22	40
	%	100	100	100	100	74.17	5.5	16.67
Zirconia	n	240	240	240	240	190	18	32
	%	100	100	100	100	79.17	7.5	13.34
Strip Crown	n	240	240	240	240	183	20	37
	%	100	100	100	100	76.25	8.34	15.42
P value		NC	NC	NC	NC	0.658		
A= Acceptable; NA= Non acceptable; D= Dropout
